# Human CD4^+^ T Cell Epitopes from Vaccinia Virus Induced by Vaccination or Infection

**DOI:** 10.1371/journal.ppat.0030144

**Published:** 2007-10-12

**Authors:** J. Mauricio Calvo-Calle, Iwona Strug, Maria-Dorothea Nastke, Stephen P Baker, Lawrence J Stern

**Affiliations:** 1 Department of Pathology, University of Massachusetts Medical School, Worcester, Massachusetts, United States of America; 2 Department of Information Services, University of Massachusetts Medical School, Worcester, Massachusetts, United States of America; 3 Department of Cell Biology, University of Massachusetts Medical School, Worcester, Massachusetts, United States of America; 4 Department of Biochemistry and Molecular Pharmacology, University of Massachusetts Medical School, Worcester, Massachusetts, United States of America; Saint Louis University, United States of America

## Abstract

Despite the importance of vaccinia virus in basic and applied immunology, our knowledge of the human immune response directed against this virus is very limited. CD4^+^ T cell responses are an important component of immunity induced by current vaccinia-based vaccines, and likely will be required for new subunit vaccine approaches, but to date vaccinia-specific CD4^+^ T cell responses have been poorly characterized, and CD4^+^ T cell epitopes have been reported only recently. Classical approaches used to identify T cell epitopes are not practical for large genomes like vaccinia. We developed and validated a highly efficient computational approach that combines prediction of class II MHC-peptide binding activity with prediction of antigen processing and presentation. Using this approach and screening only 36 peptides, we identified 25 epitopes recognized by T cells from vaccinia-immune individuals. Although the predictions were made for HLA-DR1, eight of the peptides were recognized by donors of multiple haplotypes. T cell responses were observed in samples of peripheral blood obtained many years after primary vaccination, and were amplified after booster immunization. Peptides recognized by multiple donors are highly conserved across the poxvirus family, including variola, the causative agent of smallpox, and may be useful in development of a new generation of smallpox vaccines and in the analysis of the immune response elicited to vaccinia virus. Moreover, the epitope identification approach developed here should find application to other large-genome pathogens.

## Author Summary

Although the routine use of vaccinia virus for vaccination against smallpox was stopped after eradication of this disease, there is a possibility for an accidental or intentional release of this virus. In response to this challenge, vaccination of at least emergency personnel has been suggested. However, adverse reactions induced by the smallpox vaccine have had a negative impact in the success of this program. For these reasons development of new smallpox vaccines is a public health priority. Identification of strong helper T cell epitopes is central to these efforts. However, identification of T cell epitopes in large genomes like vaccinia is difficult using current screening methods. In this work, we develop a new computational approach for prediction of T cell epitopes, validate it using epitopes already identified by classical methods, and apply it to the prediction of vaccinia epitopes. Twenty-five of 36 peptides containing predicted sequences were recognized by T cells from individuals exposed to vaccinia virus. These peptides are highly conserved across the orthopox virus family and may be useful in development of a new generation of smallpox vaccines and in the analysis of the immune response against vaccinia virus.

## Introduction

Immunization with vaccinia virus elicits long-lasting cellular and humoral immune responses in humans and in animal models (reviewed in [1]). A main component of the protective immune response elicited by this virus are neutralizing antibodies [2]. The importance of antibodies in immunity to poxviruses has been shown by passive transfer of antibodies in rodent and primate models challenged with variola virus orthologs [3,4]. B cell–deficient mice challenged with ectromelia, an Orthopoxvirus member of the same genus as the human smallpox pathogen variola, do not recover from a primary infection despite a strong CD8^+^ T cell response [5], suggesting that antibodies are an obligatory requirement for recovery of a primary poxvirus infection [3,6]. Protective antibody responses to poxvirus in mice seem to be T cell dependent [7] and require, in addition to B cells, major histocompatibility complex (MHC) class II molecules and CD40 during a secondary infection [6]. CD4^+^ T cells are also required for the generation of optimal anti-vaccinia CD8^+^ T cell responses [8]. Since protective antibody responses to poxvirus could also be elicited by immunization with single or multiple proteins in mice and in primate models [9–12], or by transfer of monoclonal or polyclonal antibodies to defined protein components [10,13,14], development of subunit vaccines would appear to be feasible and will require the characterization of CD4^+^ T cell epitopes capable of generating long-lasting antibody responses. Although human polyclonal CD4^+^ T cell responses to vaccinia virus have been documented [7,15,16], only recently have vaccinia-specific CD4^+^ T cell epitopes been reported, by Tang et al. [17], Jing et al. [18], and Mitra-Kaushik et al. [19] in humans and by Moutaftsi et al. [20] in a mouse model. Tang et al. identified three CD4^+^ T cell epitopes in the A27L protein by screening with a series of overlapping peptides covering the entire protein sequence [17]. Jing et al. followed a more comprehensive approach by screening a vaccinia genomic library that resulted in the identification of 44 separated antigenic regions of various sizes [18]. Mitra-Kaushik and collaborators approached the identification of CD4^+^ T cell epitopes in vaccinia by screening a set of 36 peptides predicted by the computational approach (described here), resulting in the identification of the first two cytotoxic HLA-DR1-restricted CD4^+^ T cell epitopes in vaccinia. In the animal model, Moutaftsi et al. screened 2,146 peptides and identified 14 epitopes restricted by the MHC molecule I-A^b^. The two large-scale screenings, by Jing et al. in humans and by Moutaftsi et al. in mice, suggest that the CD4^+^ T cell response to vaccinia virus, like the CD8^+^ T cell response, is directed against a large number of proteins. Interestingly, there is a little overlap in the T cell epitopes mapped in these studies.

Computational approaches can be used to reduce the number of potential CD4^+^ T cell epitopes to be tested. The mode of interaction of peptide antigens with class II MHC proteins is well understood from structural studies of MHC–peptide complexes and from biochemical investigation of the interaction [21], and several algorithms have been presented to predict peptide binding to particular class II MHC proteins. Most of these are based on position-specific scoring matrices or peptide binding “motifs”, which evaluate the probability of peptide binding and/or presentation by assigning a value for each amino acid at each position in a nine- or ten-residue binding frame. This approach is based on the observation that class II MHC-bound peptides adopt a conserved extended conformation with peptide side chains at certain positions binding independently into allele-specific pockets in the binding site [21]. Values for the relative contribution of each amino acid side chain at each position in a bound peptide have been evaluated by quantitative binding assays [22,23], library screening approaches [24,25], and analysis of the pool of endogenously bound peptides [26,27]. Non-matrix-based neural network and hidden Markov model binding prediction algorithms also have been presented [28–30]. However, progress in identifying class II MHC-restricted CD4^+^ T cell epitopes has been limited by the relatively poor prognostic ability of available peptide binding prediction algorithms, and the consequent need to screen large sets of potential epitopes. Epitope prediction algorithms for class II MHC proteins usually are estimated to be only approximately 50% accurate [31–33]. This low prediction ability has led many investigators to forego epitope prediction entirely, and instead test a comprehensive series of overlapping peptides completely covering the protein(s) of interest. For vaccinia this would require analysis of >3,000 peptides.

In this work we have combined aspects of previous epitope prediction approaches, and developed an efficient procedure for identifying potential HLA-DR-restricted CD4^+^ T cell epitopes. We observed interferon-γ (IFN-γ) responses to 25 out of 36 peptides predicted to contain CD4^+^ T cell epitopes. T cells recognizing these peptide sequences were observed in blood samples obtained from donors many years after exposure to the vaccinia virus, and some were expanded in response to booster vaccination, suggesting that these cells contribute to the long-lasting memory T cell response elicited by vaccinia virus [16]. In addition, most of the epitopes were conserved across many Poxviridae species, including variola. Eight peptide sequences were recognized by multiple vaccinia-exposed donors, making them ideal candidates for tracking immunity elicited by vaccination or for inclusion in subunit vaccines for this important virus family.

## Results

### Evaluation of Epitope Prediction Algorithms

Although several algorithms have been described for prediction of HLA-DR-restricted T cell epitopes, these have not been comprehensively evaluated nor compared for their relative predictive ability (a comparison of two algorithms has appeared [31]). One complication to such evaluation is that the epitope prediction algorithms generally consider only the nine to 11 residues in contact with the MHC, whereas most T cell epitope identification studies use longer peptides, within which the actual MHC binding register is not known. For evaluation of the available DR-restricted T cell epitope prediction algorithms, we selected as test antigens a series of 18 well-characterized T cell epitopes restricted by HLA-DR1 (DRB1*0101), using the Syfpeithi [33] and IEDB [34] T cell epitope databases. The protein sources for these epitopes include viral, bacterial, and tumor antigens, ranging in length from ∼200 to >3,000 residues. HLA-DR1, a common human class II allotype, has been the focus of much biochemical characterization, and for four of the test epitopes, the peptide binding register has been determined unambiguously by published crystallographic, biochemical, and peptide truncation studies (Table 1). For the other epitopes, the binding register was not as precisely defined, having been localized to a short 11- to 18-residue minimal peptide sequence by published truncation or mutagenesis studies (Table 1). In every case we required that the epitope peptide had been observed to be immunodominant or co-immunodominant among other potential epitopes derived from the same protein, by analysis of an overlapping peptide series or by characterization of naturally processed peptides.

**Table 1:**
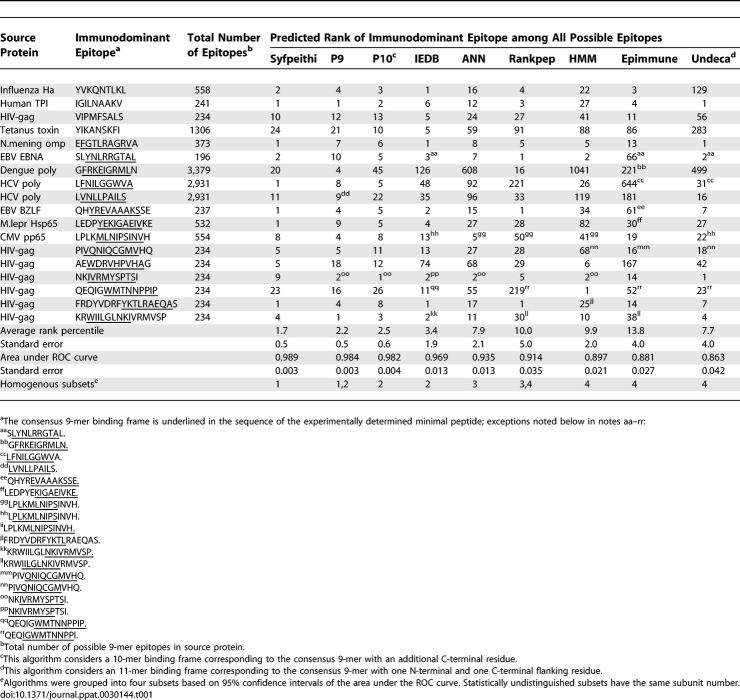
Comparison of Epitope Prediction Algorithms Using Known DR1-Restricted T Cell Epitopes

We evaluated nine prediction algorithms for their ability to predict these test epitopes. These algorithms span the variety of approaches applied to this problem, including position-specific scoring matrices derived from in vitro peptide binding data (“P9”, “P10”, “Epimmune”, “IEDB”), from random peptide library screening (“undec”), from characterization of endogenously processed peptides (“Syfpeithi”), and from bionformatic analysis of known T cell epitopes (Rankpep), and also non-matrix-based artificial neural network (“ANN”) and hidden Markov model (“HMM”) algorithms (Table 1). Most algorithms considered potential nine-residue binding frames, but one considered ten residues (P10) and one 11 (undec). For each algorithm, we scored every potential epitope present in the protein of interest, and compared these predictions to the actual immunodominant T cell epitopes found experimentally. The rank of the immunodominant epitope(s) among all the potential epitopes present in each protein is shown in Table 1. Each of the algorithms tested was successful in predicting the experimentally observed T cell epitopes. The performance of the algorithms varied, as shown by the average rank percentile values shown in Table 1. For the most efficient algorithms, the scores of the experimentally determined epitopes were found in the top ∼1%–2% of all potential binding frames (Table 1). However, even for the least efficient algorithms, the immunodominant epitopes generally were found within the top 10% (Table 1).

In addition to the ranking analysis, which evaluates the ability of the algorithms to identify the actual immunodominant epitope among the top scoring epitopes, we also performed receiver-operating characteristic (ROC) analysis [35,36], which evaluates the prediction of true positives versus false positives as the discrimination threshold is varied [37]. ROC curves were determined for each algorithm, treating the entire set of protein antigens and known immunodominant epitopes as a single test set (Figure S1). The area under the ROC curve is a measure of the probability that a randomly selected true positive will have a higher predicted score than a randomly selected true negative [37]; these values are shown in Table 1. As in the ranking analysis, each of the algorithms was able to predict the observed immunodominant epitopes far better than a random prediction (AUC ROC = 0.5). Similar trends were observed in the relative predictive power of the different algorithms as measured by ranking or ROC analysis (Table 1).

Based on ranking and ROC analysis, we selected the two top scoring algorithms (P9 and Syfpeithi) for further work. The P9 algorithm is based on an approach pioneered by Sinigaglia, Hammer, and colleagues, which uses the results of quantitative peptide binding assays for a series of single amino acid substitutions in test peptides, to predict relative MHC–peptide binding affinities [23,38]. We used a modification of the full nine-residue motif originally determined for HLA-DR4 [23] by incorporating optimized side-chain preferences for binding into the P1, P4, P6, and P9 pockets of HLA-DR1 [25] (see Materials and Methods for details). We will refer to the values from this prediction method as the “predicted binding score”. The Syfpeithi algorithm, developed by Rammensee, Stevanović, and colleagues, relies on an analysis of a database of naturally processed peptide sequences found associated with particular MHC proteins, to predict antigen processing and presentation [33]. We used the processing and presentation prediction motifs as implemented in the Syfpeithi server (http://www.syfpeithi.de/). We will refer to the values from this method as the “predicted antigen presentation score” (although the libraries of naturally processed peptides used in this approach reflect both binding and processing). The P9 and Syfpeithi algorithms also have the advantage of being derived from datasets independent of published T cell epitope work, including the epitopes analyzed in Table 1. We reasoned that combining scores from algorithms based on independent data sets would complement the deficiencies in each approach and would maximize the overall predictive power of epitope prediction. In this combination approach, we considered epitopes that were scored highly by both algorithms. We considered using a single combined P9-Syfpeithi score, but instead we used a two-dimensional plot analysis, so that potential epitopes scoring highly in one algorithm but poorly with the other could be easily identified and rejected.

**Figure 1:**
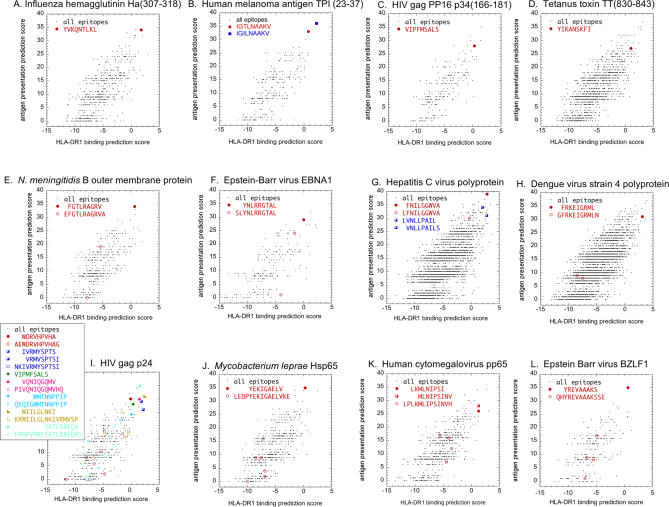
Predicted Binding and Antigen Presentation Scores for Known HLA-DR1-Restricted T Cell Epitopes (A–D) T cell epitopes for which the MHC-binding register has been definitively established. Filled circles (red) show 9-mer binding frames established by crystallographic analysis or truncation and alanine scanning mutagenesis, black dots indicate all possible 9-mer epitopes present in the entire proteins. (A–C) Binding frame identified by crystallographic analysis. (A) HA(306–318) from influenza virus heamagglutinin (A/New AYork/383/2004(H3N2)) [47]; (B) mutated TPI(23–37) from human triose phophate isomerase containing Ile instead of Thr at position 28 [77,78], with filled blue square indicating mutated peptide recognized as a tumor antigen; (C) PP16 p24(161–181) from HIV-1 strain NY-5 gag p24 protein; (D) TT(830–843) from tetanus toxin [46]; (E–H) T cell epitopes identified by overlapping peptides for which a small minimal peptide epitope has been characterized by truncation analysis. For each minimal peptide epitope, all of the possible 9-mer binding frames are shown as open circles, with the likely MHC binding frame, identified as a 9-residue sequence starting with a hydrophobic residue near the N-terminus of the peptide [23,38,70], indicated by a filled symbol. (E) N. meningitidis outer membrane porin A protein (91–108) [79]; (F) EBV nuclear antigen EBNA-1([78–88) [80]; (G) hepatitis C virus strain LIV23 polyprotein. Two epitopes have been characterized: NS4(1809–1817) and NS4(1879–1888) [81], shown in red circles and in blue squares, respectively. For the NS4 epitope, the minimal 10-mer peptide contains two likely binding frames, indicated by half-filled squares. (H) Dengue virus type 4 virus D4V capsid(84–92) [82]. (I–L) T cell epitopes identified by overlapping peptide analysis with larger minimal peptides. As in (E–H), presumptive binding frames are indicated by solid symbols with other potential epitopes from the same minimal peptide shown with open symbols. (I) HIV-1 (strain HXB2) p24 gag epitopes: AEWDRVHPVHAG(210–221) [83] in red circles; NKIVRMYSPTSI(271–282) [83] in blue squares, as in (G) two possible binding frames are indicated with half-filled squares; HIV-1 (strain NY5) p24 gag epitope PEVIPMFSALSEGATP(167–182) in green diamonds [84], for this epitope the binding frame is known exactly as shown in (C); PIVQNIQGQMVHQ(133–145) in magenta up-facing triangles; QEQIGMTNNPPIP(244–256) [83] in cyan down-facing triangles; KRWIILGLNKIVRMVSP(264–280) [85,86] in brown left-facing triangles; FRDYVDRFYTLRAEQAS(294–311) in aquamarine right-facing triangles [86,87]. (J) Mycobacterium leprae heat shock protein hsp65(61–75) [88]. (K) Human cytomegalovirus (strain AD169) pp65(115–127) [89], as above with two potential binding frames shown in half-filled symbols, (L), EBV BZLF-1 protein(198–210) [90,91].

We evaluated the combination approach in predicting the same set of well-characterized HLA-DR1 T cell epitopes described above. Dot plots of predicted peptide binding and antigen presentation scores for each potential 9-mer epitope are shown in Figure 1, with the actual epitope(s) present in each protein shown by a solid symbol. In cases where the binding frame within the known epitope was not completely determined by crystallography or mutagenesis (Figure 1E–1L), open symbols show the other potential binding frames. In each case, the actual T cell epitopes were found in the extreme upper-right region of the plot of all scores. These results suggested that a combined predicted binding and presentation approach would be useful in identifying MHC class II-restricted T cell epitopes from vaccinia virus.

### Identification of Potential Vaccinia Epitopes

**Figure 2:**
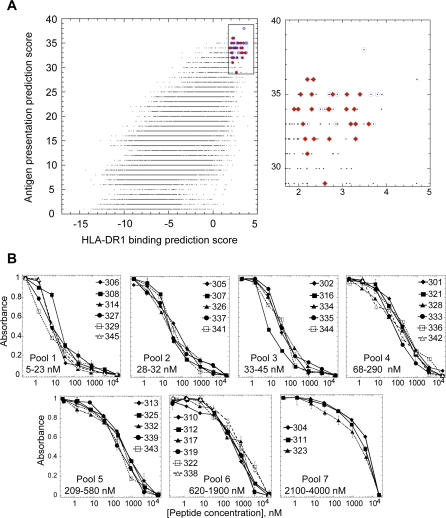
Prediction of Vaccinia-Derived HLA-DR1-Binding Peptides and Experimental Validation of Binding Affinity (A) HLA-DR1 predicted binding score and predicted antigen presentation score for each 9-mer potential peptide epitope are indicated by dots. Circles show peptides selected for further analysis (Table 1), filled circles indicate peptides for which T cell responses were observed. Inset shows high-scoring region. High-scoring peptides that were not included in the study because of synthetic difficulty are indicated by x. (B) Competition binding assay to evaluate MHC–peptide affinity. The ability of various concentrations of unlabelled MVA peptide to compete with biotinylated test HA peptide for binding to recombinant soluble HLA-DR1 was determined. Values shown (error bars) represent average (standard deviation) of three independent binding experiments. IC_50_ values were determined by fitting to a competition binding equation. Peptides were grouped as indicated into seven pools based on observed IC_50_ values.

We used the combined P9 binding and Syfpeithi presentation algorithms to predict DR1-restricted T cell epitopes from the modified vaccinia Ankara (MVA) [39] strain of vaccinia virus. Figure 2A shows a plot of the predicted P9 peptide binding and Syfpeithi antigen presentation scores calculated for each of the 47,658 different 9-mer peptide sequences present in predicted open-reading frames of the genomic sequence. Thirty-six 9-mer sequences with high scores in both algorithms were selected for continued analysis (Figure 2A, inset). These cutoffs represent the top 0.4% and 1.1%, respectively, of all the predicted scores for the P9 and Syfpeithi scores. For each sequence, a 21-mer peptide was synthesized containing the 9-mer sequence of interest flanked by several residues on each side to allow for productive T cell interaction with residues outside the direct MHC–peptide contact region [40] (Table 2). HLA-DR1 binding to each of these peptides was evaluated by a competition assay (Figure 2B; Table 2). For immunogenicity analysis, the peptides were grouped according to the experimental IC_50_ values into seven pools of three to six peptides (Figure 2B; Table 2). In this manner we hoped to minimize inter-peptide competition within a pool that could result in poor responses to peptides with lower affinities.

**Table 2:**
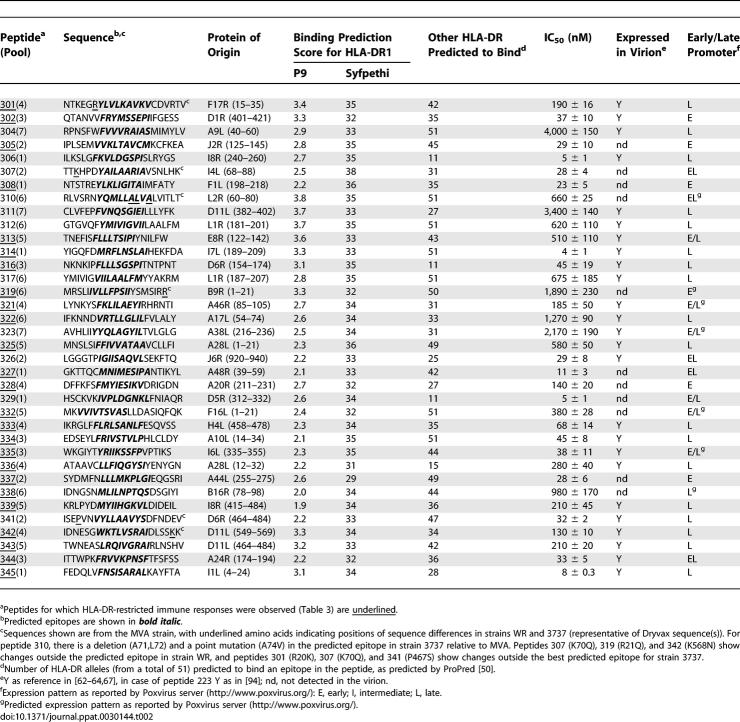
Predicted HLA-DR-Restricted T Cell Epitopes from Vaccinia Virus

**Table 3:**
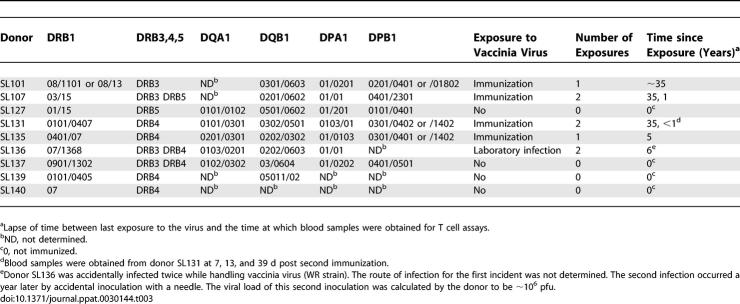
Haplotype of Donors and Exposure to Vaccinia Virus

### Analysis of CD4^+^ T Cell Responses in a Vaccinated HLA-DR1 Donor

**Figure 3:**
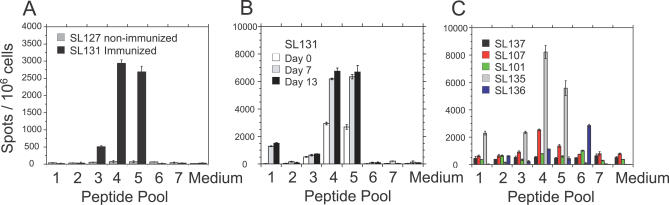
TCLs Raised from Vaccinia Immune Donors Recognize Pools of Predicted HLA-DR1-Binding Peptides (A) IFN-γ ELISPOT response of vaccinia-specific TCLs from DR1^+^ donors to pools of predicted peptides. TCL were generated from PBMCs obtained from a non-immunized (gray bars) or immunized (dark bars) DR1^+^ donor by stimulation with a crude preparation of heat-inactivated vaccinia virus. After 15 to 20 d of in vitro expansion, cells were tested in an IFN-γ ELISPOT assay using autologous PBMCs as APCs and the peptide pools as antigen. T cell assays were performed in cRPMI+10% human serum (see Materials and Methods). (B) Kinetics of the IFN-γ ELISPOT response in the immunized DR1 donor after a boosting immunization: shown are the T cell responses to the peptide pools for TCLs generated from blood samples obtained at the indicated time points. (C) Recognition of peptide pools by TCLs generated from PBMCs obtained from five non-DR1 donors: three immunized (SL101, SL107, and SL135), one accidentally infected with the vaccinia virus WR strain (SL136), and one non-immunized (LS 137).

To evaluate the presence of actual class II T cell epitopes in the predicted set of peptides, we initially studied the T cell responses in two HLA-DR1 (DRB1*0101) donors, one previously immunized against smallpox approximately 35 years earlier (SL131) and one non-immunized and presumably naïve with respect to vaccinia virus (SL127) (Table 3). From previous studies of the human CD4^+^ T cell response to vaccinia, we expected that vaccinia-specific T cells would be present at low frequency in an immunized donor [16], but would be capable of antigen-driven expansion in vitro for many years after exposure to the virus [16,41–43]. Thus, we generated T cell lines (TCLs) from peripheral blood mononuclear cell (PBMC) preparations from these donors by in vitro expansion with a heat-inactivated lysate of CV-1 cells infected with Dryvax vaccinia virus as a source of vaccinia antigens [19]. Dryvax was the prevalent vaccine formulation used for vaccination in the US up to the eradication of smallpox worldwide in 1977. Figure 3A shows the IFN-γ ELISPOT response of TCLs from the two DR1 donors to the pools of peptides presented in Table 2. There is a striking difference in the recognition of peptide pools by these two volunteers. The numbers of IFN-γ-secreting cells responsive to peptide pools 3, 4, and 5 in a TCL raised from the immunized donor (dark bars) are five to 50 times larger than the corresponding numbers in a line raised from the non-immunized donor (gray bars).

The responses to peptide pools 3, 4, and 5 observed for the immunized donor appear to represent a pool of long-lasting memory T cells persisting for >35 years after Dryvax immunization. If these responses were elicited by vaccination, they should be boosted by a second re-immunization [43]. An analysis of the IFN-γ response of TCLs raised from PBMC samples of the immunized donor obtained on days 7 and 13 after a second Dryvax dose demonstrated that responses to pools 3, 4, and 5 are boosted by a second immunization and also revealed T cell specificities to pool 1 and a weak response to pools 2 and 7 not observed in the long-lasting memory pool apparent before boosting (Figure 3B). Overall, re-vaccination of this individual resulted in approximately a 2-fold increase in the number of IFN-γ-secreting cells recognizing peptides in pools 3, 4, and 5, when compared to the numbers observed in the TCL prior to boost, and also resulted in a broadening of the response to include recognition of peptide pools not observed prior to boost.

### Extension to Other HLA-DR Haplotypes

The identification of peptides presented by multiple MHC class II haplotypes is an important goal of epitope discovery efforts, and in this case could help efforts to induce and track poxvirus immunity in a larger segment of the populations. In previous work, several HLA-DR-restricted epitopes from a variety of proteins have been shown to be presented by multiple class II MHC proteins [44–46]. This is a reflection of the similarity of peptide binding preferences within the HLA-DR family of alleles, all of which share a conserved alpha subunit that contributes much of the surface area of the important P1 and P9 pockets [47]. Moreover, HLA-DR1 (DRB1*0101) is considered an exemplar of a large fraction of the entire HLA-DR family, as most HLA-DR1-binding peptides also bind well to other HLA-DR alleles [48,49]. For example, each of the peptides in Table 1 is predicted by a matrix-based binding algorithm [50] to bind to many different alleles in the HLA-DR family, with a minimum of 11 predicted binding alleles and an average of 37 out of 51 total HLA-DR alleles considered in the analysis (Table 2).

To evaluate the ability of the peptide pools to be recognized by vaccinia-specific T cells in the context of other HLA-DR alleles, we raised TCLs as above from blood samples of five individuals with other haplotypes (Table 3). This second group was highly heterogeneous with respect to their MHC haplotypes, age, and exposure to the virus. Volunteers SL101 and SL135 were immunized once and volunteer SL107 twice, volunteer SL136 was accidentally infected twice while working with the Western reserve strain of vaccinia virus (WR), and volunteer SL137 had no previous exposure to vaccinia virus. TCLs from these donors were tested for their ability to recognize the peptide pools as described above. In spite of the variability in this group, TCLs raised from each of the vaccinia exposed donors recognize pools 4 and 5 (Figure 3C); these pools were recognized best by the immunized DR1 donor (Figure 3A and 3B). Pool 3 was recognized by two of the three immunized donors and by the infected donor (SL136). Pools 2 and 6 were also recognized by three of four vaccinia-exposed donors. Interestingly, only in the infected donor did we observe a significant response to pool 6. Pool 1 was recognized by T cells from only one of the immunized donors, and pool 7 induced a higher number of IFN-γ cells in the non-immunized donor as observed also in the HLA-DR1 donors. Thus, despite the differences in MHC haplotype, the vaccinia-exposed donors followed a similar pattern of reactivity described above for the immunized DR1 donors. The magnitude of the responses do not seem to correlate with the number of doses in this small cohort of volunteers, since donor SL135, immunized once, recognizes at a higher frequency a larger number of pools than does donor SL107, immunized twice (Table 3).

### Deconvolution of Peptide Pools and Identification of Immunodominant CD4^+^ T Cell Epitopes

**Figure 4:**
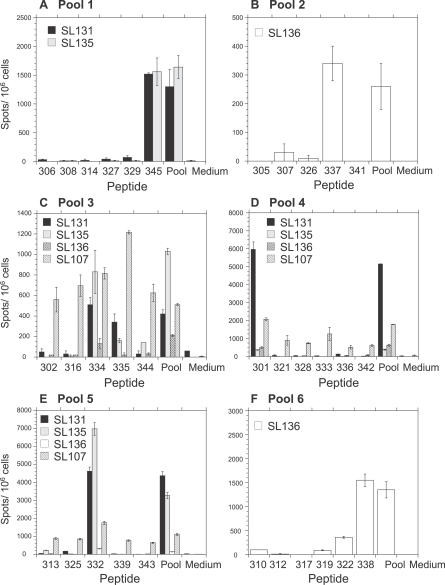
Fine Specificity of TCLs Generated in Vaccinated and Infected Donors Fine specificities were determined by IFN-γ ELISPOT using as antigen individual peptides included in a given pool. (A) Peptide pool 1; (B) peptide pool 2; (C) peptide pool 3; (D) peptide pool 4; (E) peptide pool 5; and (F) peptide pool 6. Background responses (wells in which peptide was not added) were on average less than 22 spots per 10^6^ cells (range 2 to 34).

To define the particular peptides recognized in a given pool, TCLs from the responding donors were tested separately with each of the individual peptides present in the peptide pools (Figure 4). The overall analysis of the response to individual peptides indicates that several of the peptides are recognized by TCLs from multiple donors with roughly similar patterns despite the differences in MHC haplotypes, but also that the donors differ in the breadth of their responses against these peptides.

#### Pool 1.

This pool contains the peptides with the highest relative affinity for DRB1*0101 molecules and was recognized at significant levels by two of the four immunized donors, the DR1^+^ donor SL131 and donor SL135. We therefore studied TCLs from these two donors and found that T cell responses in this pool are directed mainly to peptide 345 (Figure 4A). A second in vitro stimulation of these TCLs with the crude preparation of heat-inactivated vaccinia virus reveals IFN-γ responses in SL131 to peptides 308, 314, and 327 (Figure S2).

#### Pool 2.

Peptide pool 2 was recognized at a relatively low level by some of the immunized volunteers and by the infected donor. Since the infected donor's (SL136) response was three to six times higher than that of the other three volunteers, we mapped the response only in SL136. In this case the response to the peptide pool is directed to peptide 337 (Figure 4B). The remaining peptides in this pool do not induce IFN-γ responses even after a second in vitro stimulation of the TCL (Figure S2).

#### Pool 3.

This pool was consistently recognized by multiple vaccinia-exposed donors. T cells from four donors were used to map the response in this pool. Peptides 334 and 335 were recognized by T cells from four and three donors, respectively (Figure 4C). The remaining peptides, 302, 316, and 344, were recognized at relatively high levels only by donor SL107. Two of these peptides, 302 and 316, were weakly recognized by SL131 after a second in vitro expansion (Figure S2). Although peptide 335 was tested at the same concentration in isolation and in the pool, the numbers of cells of volunteer SL107 responding to this peptide is greater than the number of cells responding to the pool, suggesting that some type of peptide competition is occurring in the pooled peptide experiments. This effect is also observed in responses to peptides from pools 4 and 5.

#### Pool 4.

This pool is recognized by all of the immunized donors. For two of the immunized donors and the infected donor, peptide 301 accounts most of the response in this pool (Figure 4D). As before, donor SL107 recognize all the peptides in this pool, although significantly lower numbers of T cells are observed when compared with the numbers in response to peptides in pool 3 (Figure 4C). Donor SL135 has a small, but positive response to peptide 342 and a weak response to peptide 336. This last peptide is also weakly recognized by donor SL131. A second in vitro expansion of the SL131, SL135, and SL136 reveals responses to peptides 321, 336, and 342 in the TCL from donor SL135.

#### Pool 5.

This pool is recognized by all of the vaccinia-exposed donors and consequently we evaluated the IFN-γ response from volunteers SL131, SL107, SL135, and SL136. In this case, peptide 332 accounts for most of the response in all the donors (Figure 4E). A weak response to peptide 313 is observed in SL135 TCL, while SL107 TCL presents weak responses to this and the remaining peptides in the pool. As before, a second in vitro expansion confirmed some of the weak responses in the pool. In this manner, IFN-γ responses to peptide 313 are observed in SL131 and to peptide 325 in donors SL131 and SL135 (Figure S2).

#### Pool 6.

This peptide pool is recognized strongly by the infected donor SL136 and at a low level by two of the immunized volunteers. We consequently mapped the response in SL136 (Figure 4F). The response to this pool is directed mainly to peptide 338, with a minor component to peptides 319 and a weak response to peptide 322. Responses to peptide 332 were confirmed after a second in vitro expansion of the TCLs (Figure S2).

In summary, we have observed that with the exception of pool 3, in which three peptides are recognized by multiple donors at relatively high levels, the responses in the pools were dominated mainly by a single peptide: peptide 345 in pool 1, peptide 337 in pool 2, peptide 301 in pool 4, peptide 332 in pool 5, and peptide 338 in pool 6. Responses to the remaining peptides in these pools were significantly weaker or absent. These immunodominant peptides exhibited a range of affinities in our competition binding assays, consistent with the idea that other factors besides peptide–MHC affinity influence immunodominance patterns [51–53].

### Validation of the Approach Using Additional Peptide Pools

**Figure 5:**
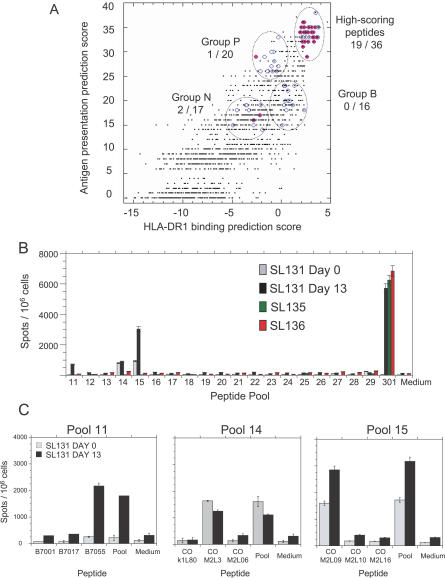
Validation of the Prediction Algorithm and Recognition of Vaccinia Peptides That Fell outside the Boundaries of the High Score Region for Binding and Presentation (A) HLA-DR1 predicted binding and predicted antigen presentation scores are shown for each 9-mer potential peptide epitope from the vaccinia genome, Open circles indicate peptides tested and closed red circles indicate peptides for which T cell responses were observed. This graph includes the 36 peptides presented in Figure 2 and the set of 53 peptides used to validate the prediction algorithm. Peptides are clustered according to their binding and presentation scores into groups H, P, B, and N. Only three peptides outside the H region are recognized by T cells. (B) IFN-γ ELISPOT responses of SL131, SL135, and SL136 TCLs to pools of peptides included in P, B, or N regions. (C) Deconvolution of the IFN-γ response of SL131 TCL day 0 (gray bars) and day 13 (solid bars) to positive pools.

To validate the combination binding/presentation prediction approach used in the initial selection of epitopes for testing, we utilized a second set of 53 synthetic peptides with scores that fell outside the boundaries of the high scoring region (group H) of the predicted peptide binding and presentation plot (Table S1; Figure 5A), and evaluated these for recognition by twice-stimulated TCLs from SL131, SL135, or SL136 donors. Peptides were selected from three regions of the plot: high antigen presentation prediction score (Syfpeithi > 25, group P), high HLA-DR1 binding predicted score (P9 > 0, group B), and both low predicted binding and low presentation scores (Syfpeithi < 20 and P9 < −1.9, group N). The peptides, which ranged from 13 to 20 residues in length and derived from 19 different vaccinia proteins, were tested in pools of three peptides, with positive responses deconvoluted as described above for the original set of peptides (Figure 5B and 5C). None of the 16 group B peptides were recognized by TCLs from SL131, SL135, or SL136 donors. One of the 17 group P peptides (B7055) and two of the ten group N peptides (COM2L03 and COM2L09) were recognized by TCL from DR1^+^ donor SL131. Volunteers SL135 and SL136 do not recognize any peptide in this new set. These values can be compared with much more efficient identification of the peptides from the original set of peptides scoring highly in both algorithms, for which 19 of 36 peptides were recognized by TCLs from the same donors (Table 4). Moreover, of the three low-scoring peptides recognized by SL131 TCL, only one (B7055) bound with appreciable affinity to HLA-DR1 in a competition assay (IC_50_ 11 nM, unpublished data).

**Table 4:**
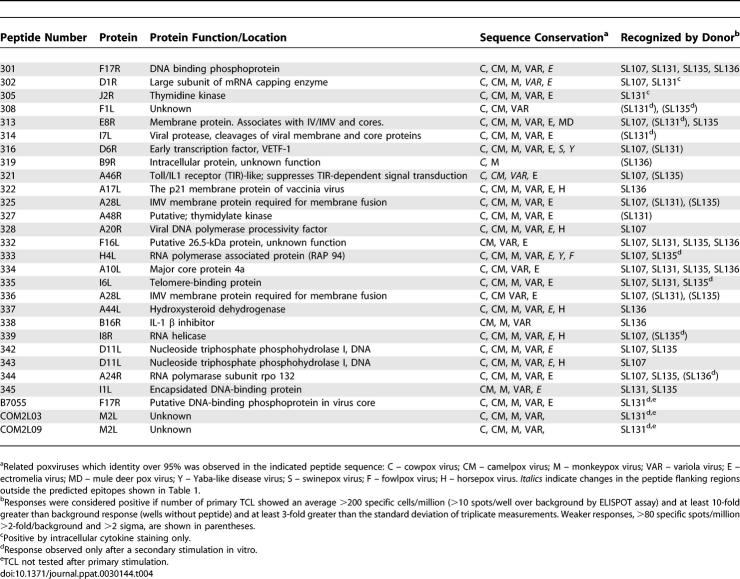
Vaccinia-Derived T Cell Epitopes

Using both the original high-scoring peptides and the low-scoring group P, B, and N peptides, the predictive power of the combined binding and prediction algorithm can be assessed. Examination of the distribution of observed epitopes in the binding and presentation dot plot (Figure 5A) reveals that the combination of both binding and prediction scores is more indicative of peptide recognition by T cells than is either score used alone. This point is substantiated by a variety of descriptive statistics calculated for generous cutoff scores of P9 > 0 and Syfpeithi > 25 (which each select ∼5% of all potential epitopes) (Table S2). In each case, the combination scores were narrowly higher than either P9 or Syfpeithi alone. Similar results were observed for other choices of cutoff scores (not shown). The area under the ROC curve takes into account all possible cutoff criteria, and for this statistic as well the combination scores better than either P9 or Syfpeithi alone (Table S2). The combination P9 > 0 and Syfpeithi > 25 also scored narrowly higher than single combined values based on the product or the sum of P9 and Syfpeithi values (Table S2). Thus, the epitope identification approach described here efficiently identifies T cell epitopes, with the combination of both binding and prediction algorithms superior to the use of either alone.

### MHC Restriction

The peptide sequences in the present study were selected based on predicted binding and presentation scores for HLA-DRB1*0101 molecules. We observed responses to these peptides in individuals that do not carry this allele. In order to investigate if DRB1*0101 (DR1) was in fact presenting the vaccinia peptide in SL131, a DR1^+^ donor, we studied the presentation of peptides 301, 302, 305, 325, 332, 334, and 335 by peptide-pulsed homozygous LG2 Epstein–Barr virus (EBV)-B cells to SL131 TCL day 13 (Figure S3). LG2 cells and SL131 share only DRB1*0101 and DQB1*0501 (Figure S4), and we observed that all the peptides were presented by LG2 cells. Antibodies to DR, but not anti-MHC class I antibodies, inhibit the presentation of these peptides, suggesting presentation by MHC DRB1*0101 (DR1). Restriction of peptides 301, 325, and 332 was corroborated by using additional antigen-presenting cells (APCs) that share other class II molecules with SL131 (Figure S4). Cell lines lacking DR1 exhibited a significantly reduced presentation, indicating that responses to peptides 301, 325, and 332 in donor SL131 are restricted by DR1. In addition, peptide 325 seems to be also presented to T cells in this TCL in the context of other class II molecules, since EBV-B 9273 cells sharing DRB4*, DQB1*0501, and DPB1*0301 with donor SL131 also were able to activate T cells.

### Detection of Vaccinia-Specific T Cells in Peripheral Blood Samples

**Figure 6:**
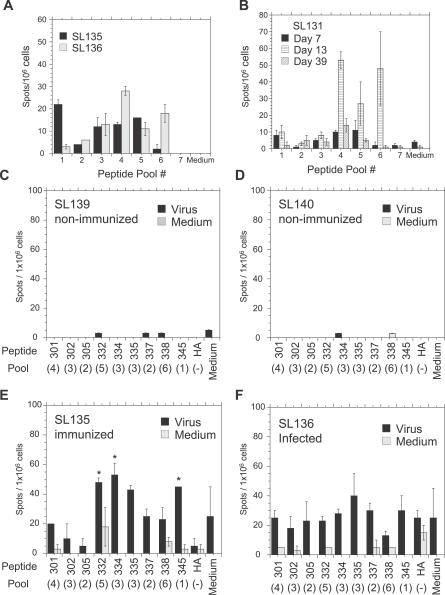
Ex Vivo IFN-γ Response of T Cells in Peripheral Blood to Vaccinia Peptides Response in PBMCs isolated from vaccinia-exposed donors and from non-exposed donors to pools of vaccinia peptides or individual peptides, measured by IFN-γ ELISPOT. (A) IFN-γ responses of PBMCs in an immunized donor (SL135) and in an infected donor (SL136). No spots were observed in control wells (cRPMI+10% HS, no peptide). (B) Kinetics of the IFN-γ response after a boosting immunization of donor SL131, a DR1 donor. Spots for control wells (cRPMI+10% HS, no peptide) day 7 4+/−1, day 13 1+/−1, and day 39 0+/−0 (C–F) IFN-γ responses in PBMCs after depletion of CD8^+^ T and overnight incubation in cRPMI+10% HS (gray bars) or incubation in cRPMI+10% HS supplemented with a crude preparation of heat-inactivated vaccinia virus (solid bars). (C, D) non-immunized donors SL139 and SL140. (E, F) vaccinia-immunized donors SL135 and SL136. Spots in control wells (Medium, no peptide) 25+/−20. Statistically significant responses (*p* <0.05) indicated by an asterisk.

For tracking immunity induced by vaccination, it would be useful to be able to detect T cell responses directly in circulating PBMCs without the need for in vitro expansion and extended culture. To evaluate whether T cell responses to the vaccinia peptides were detectable directly *ex vivo* in PBMCs from vaccinia-immune donors, we used IFN-γ ELISPOT assays and the same peptide pools as before. Significant T cell responses were observed for PBMCs from both donors SL135 (immunized) and SL136 (infected) to peptides in pools 3, 4, and 5, with SL135 PBMCs also recognizing pool 1 and SL136 also recognizing pool 6 (Figure 6A). This is the same pattern as observed for the TCL raised from these same donors (Figure 3C). PBMCs were analyzed at several time points after boosting immunization for the DR1^+^ donor SL131 (Figure 6B). Responses were observed to pools 1, 3, 4, and 5, with the responses to pools 4, 5, and especially 6 increased on day 13 following the second immunization (Figure 6B). However, by day 39, PBMCs response to this pool and the others fall to their original levels at day 7. Overall, the vaccinia-specific T cells observed in the *ex vivo* ELISPOT assay represent only a small fraction of the total population of T cells present in the PBMC samples, corresponding to ∼five to 20 cells per million for the long-term memory responses and up to ∼50 cells per million shortly after re-immunization. The ELISPOT assay does not distinguish CD4^+^ and CD8^+^ T cell responses, and potentially both could contribute to the observed response. To confirm the role of CD4^+^ T cell, new samples of PBMCs were obtained, depleted of CD8^+^ T cells, and subject to a short stimulation with a crude preparation of heat-inactivated vaccinia virus (or medium alone) before IFN-γ ELISPOT analysis. For this study we obtained PBMCs from the vaccinia-exposed donors SL135 and SL136 and from two non-immunized donors, SL139 and SL140. After depletion, the number of CD8^+^ T cells in these preparations was reduced to less than 0.31% (Figure S5). Figure 6C–6F shows the responses to a set of ten peptides that includes the dominant peptides in the pools and as a negative control a peptide representing the influenza virus hemagglutinin HA peptide [54]. PBMCs from SL135 have significant responses to peptide 345 (pool 1), peptides 334 and 335 (pool 3), and peptide 332 (pool 5) (Figure 6E). T cells from donor SL136 or the non-immunized donors SL139 and SL140 do not have statistically significant responses to any of the peptides evaluated by this assay (Figure 6C, 6D, and 6F). In summary, CD4^+^ T cell IFN-γ responses to three vaccinia peptides could be observed in PBMC samples from an immunized donor immediately after booster immunization, but also many years after exposure to the virus.

## Discussion

Vaccinia virus is the central component of the smallpox vaccine, used in the only successful eradication of an infectious disease (reviewed in [55]). Concerns about the potential use of smallpox virus as a biological weapon, combined with a high morbidity rate and contraindications to immunization with vaccinia virus in some segments of the population, and recent outbreaks of monkeypox and other related poxviruses [56,57], all highlight the need for a new generation of smallpox vaccines [58,59]. For these reasons, new strains of vaccinia virus have been developed [60,61] and subunit vaccines for smallpox are in development [11,12]. However, advancement in this field is hampered by the lack of well-characterized CD4^+^ T cell epitopes required for the induction of long-lasting cellular and humoral immune responses.

We approached the challenge of identifying CD4^+^ T cell epitopes in vaccinia using an algorithm that combines independent assessments of MHC–peptide affinity [23,25] and propensity for MHC-mediated antigen presentation [33]. These two algorithms each proved highly predictive in an evaluation of 18 well-characterized HLA-DR1-restricted immunodominant epitopes. The combination approach was extremely effective in identifying vaccinia-derived CD4^+^ T cell epitopes. Of 36 potential epitopes tested, we observed IFN-γ responses to 25 peptide sequences, with strong responses in multiple donors observed for ten peptides. By comparison, in a recent study >2,000 peptides were screened to identify only 14 epitopes [20]. The 25 peptides for which we observed T cell responses were derived from 23 proteins (A28L and D11L had two epitopes each). These proteins represent a mixture of early, late, and intermediate proteins, and include proteins present in the virion as well as proteins expressed only in infected cells (Table 3).

Peptides 301 and 334, which were recognized by all of our vaccinia-immune donors, are derived from proteins F17R and A10L, respectively, proteins that have been reported to be among the very most abundant proteins in the intracellular mature virion particles [62]. A CD4^+^ T cell response to a different region of protein A10L was recently reported [18]. Peptides 342 (D11L), 343 (D11L), 344 (A24R), and 302 (D1R) are all derived from enzymes involved in nucleic acid metabolism, and which are also highly represented in vaccinia virions. Ten other epitopes also derive from proteins present in vaccinia virions (IMV), but are reported to be at lower abundance [62–64]. We used a crude lysate of infected cells as a source of vaccinia virus antigens for in vitro amplification of TCLs, and so we expected that our experimental protocol would allow identification of T cell responses directed against proteins present in viral particle and also against proteins not present in the virus but which are expressed in infected cells. Six peptides (305, 319, 328, 332, 337, and 338) derive from proteins not reported to be present in vaccinia virions. Peptide 332, derived from putative protein F16L, is recognized by all the vaccinia-exposed donors, but is not present in purified virions [62–64]. Two peptides, 337 and 338, are derived from the immunomodulatory and virulence factors A44L (hydroxysteroid dehydrogenase) [65] and B16R (IL-1 β inhibitor) [66], and also are not expected to be present in the virion. Interestingly, we observed preferential responses to these epitopes in the infected as compared to vaccinated donors. However, inter-individual variation in the T cell responses to vaccinia virus as reported by Jing et al. could also explain these observations [18]. T cell responses to peptides 306, 308, 314, and 327 were only evident after a second in vitro expansion. These peptides belong to the proteins I8R, F1L, I7L, and A48R, respectively. Only F1L, containing 306, and I7L, containing 314, are present in the virion. DR-restricted CD4^+^ T cell responses to proteins I8R and A48R have been reported to other regions of these proteins [18].

Envelope proteins A27L, A33R, B5R, and L1R have attracted attention as possible subunit vaccine candidates because antibodies against these proteins correlate with protection against viral challenge in animal models [12]. We tested two peptides derived from L1R (312 and 317), but did not observe responses in our donors. Both A33R and B5R have at least one peptide with very favorable HLA-DR1 binding prediction and antigen presentation scores that, however, fell just outside the range that we tested, and both of these proteins as well as L1R have additional peptides with scores in a slightly more generous region. Additional testing will be required to determine whether these high-scoring sequences do elicit the CD4^+^ T cell responses observed in these proteins. A27L does not contain potential epitopes that score highly by this algorithm. Although responses to A27L, A33R, B5R, and L1R were not observed among the set of peptides that we tested, we did identify T cell epitopes (peptides 322, 325, and 338) from three other proteins expected to be present on the virion membrane, A17L (p21 membrane protein), A28L (IMV membrane protein required for membrane fusion), and F16L (IL-1 β inhibitor). A17L is reported to be present in the inner of the two membranes of the IMV particles, with the N-terminus of the protein protruding to the surface of the particle, and antibodies raised against the exposed fraction of the protein neutralize vaccinia virus [67]. These proteins might be considered as possible candidates for inclusion in subunit vaccine development efforts. Finally, antibody responses have been reported for protein I1L, containing peptide 345, and protein A10L, containing peptide 334 [9]. However, there is currently no information about the possible role of these antibodies in immunity to vaccinia.

Only very recently have CD4^+^ T cell epitopes been identified in vaccinia virus [17–20]. Jing et al. [18] reported human T cell responses to 35 vaccinia proteins, and Moutaftsi et al. [20] reported murine T cell responses to 13 vaccinia proteins. In this study, we detected in total human T cell responses to 28 peptide sequences from 24 different proteins, including the peptides with low prediction scores. T cell responses defined in this study and the ones reported by Jing et al. [18] have in common only the response to three proteins, with no overlap in the actual peptide epitopes recognized. Comparison of the human T cell responses reported in our study and the murine responses reported by Moutaftsi et al. reveals a partial overlap in the T cell response to peptide 328, and a complete overlap in the response to peptides 325 and 345 in the proteins A20R, A28L, and I1L, respectively. Tang et al. [17] reported human responses to three A27L epitopes in T cells isolated from blood samples obtained after 1 month or 3 years of vaccination; no epitopes from this protein were identified in our study or in the studies of Jing et al. [18] or Moutaftsi et al. [20]. Finally, using the same set of peptides tested here, Mitra-Kaushik et al. [19] reported cytotoxic CD4^+^ T cell to D1R (MVA302) and A24R (MVA341) peptides, for which we observed robust IFN-γ responses. Whether the difference between these reports reflect individual (“private”) patterns of immunodominance, differences between the TCL and assay protocols used by the different groups, or incomplete sampling of a broad response to many different antigens, remains to be established by further work.

An important factor to consider in the characterization of T cell epitopes is the sequence conservation, since viral variation potentially can evade T cell immunity. Current vaccines induce substantial cross-protection between poxvirus family members. The 35 peptide sequences presented in this study are highly conserved among vaccinia strains, with only peptides 301 and 310 exhibiting variation within the core epitope or the immediately flanking residues (Table 1). The epitope sequences also are conserved within the larger poxvirus family (Table 3). Peptides 301, 332, 334, 335, 344, and 345, which each were recognized by strong responses in at least two of the donors in our study, are conserved in at least three poxvirus, including the human pathogens variola (all peptides) and monkeypox (all but 332). CD4^+^ T cells recognizing three of these peptides (332, 334, and 345) were observed in PBMCs from a vaccinated donor, indicating that cells with these specificities make up a significant part of the long-lasting memory pool elicited by immunization with vaccinia virus.

Our identification of highly conserved CD4^+^ T cell epitopes derived from vaccinia virus and recognized by donors of multiple MHC haplotypes could aid efforts to track cellular immunity induced by next-generation smallpox vaccines and could contribute to selection of candidate proteins for inclusion in potential subunit vaccine approaches. The epitope identification algorithm described here appears to be a significant improvement over current approaches, and could find application in prediction of class II MHC-restricted T cell responses to other large-genome viral and bacterial pathogens.

## Materials and Methods

### Peptide synthesis.

Peptides were synthesized by Genemed Synthesis using standard Fmoc chemistry and were characterized by high performance liquid chromatography (HPLC) using a Vydac-C18 reverse phase column and by MALDI-TOF mass spectrometry. All peptides were >80% pure as judged by HPLC and exhibited molecular masses consistent with the expected sequence.

### Vaccinia virus.

Vaccinia virus (Dryvax) stocks were propagated and provided by John Cruz (University of Massachusetts Medical School). This virus was expanded in CV-1 cells, titrated, and stored at −80 °C as previously reported [68]. For T cell assays and for in vitro expansion of T cells, lysate of CV-1 infected cells was treated at 60 °C for 1 h to inactivate the virus [69].

### Epitope prediction.

Translated poxvirus genome sequences were obtained from the Poxvirus Bioinformatics Resource Center (http://www.poxvirus.org/). HLA-DR1 (DRB1*0101) binding epitopes were predicted using the genomic sequences of vaccinia virus strain MVA [39]. In some cases, epitopes were compared to orthologs in the vaccinia virus strains WR and 3737. The latter strain was isolated from a vaccinia lesion following vaccination with Dryvax, and is used an exemplar of Dryvax component strain(s). Predictions for non-vaccinia protein in Figure 1 and Table 1 used sequences from the National Center for Biotechnology Information (NCBI) as indicated. Syfpeithi, P9, P10, undec, and Epimmune predictions were performed using position-specific scoring matrices and local software (C. Parry and L. J Stern, unpublished data). The Sypfeithi matrix for the prediction of antigen processing and presentation by HAL-DR1 was obtained from the Syfpeithi server (http://www.syfpeithi.de/) [33]. For the Syfpeithi algorithm, scores can potentially range from 0 to 42; for all nine-residue sequences in the MVA translated genome, the average score was 11.9 (standard deviation 7.3). The P9 matrix for prediction of peptide binding to HLA-DR1 was obtained by modification of the virtual DR1 matrix originally described by Sturniolo et al. [38]. For the dominant pocket P1 [25,70,71], a simple aromatic/aliphatic/other profile was used: Trp, Tyr, and Phe were assigned a value of 0, Ile, Leu, Val, and Met a value of −1, and all other residues assigned −5. This profile is similar to but more permissive than that incorporated into the original virtual matrix motif [38]. Profiles of the major pockets at P4, P6, P7, and P9 were retained from the virtual matrix [38]. The profiles at the minor pocket P2 and P3 positions were obtained from a full matrix originally determined for DR4 [23], since these pockets are essentially identical in DR1 and DR4. Finally, P5 and P8 profiles were set to 0 for all residues (these side chains make no or minimal contact with HLA-DR [47]). For this algorithm scores can potentially range from −16.6 to 6.4; for all nine-residue sequences in the MVA translated genome the average score was −5.3 (standard deviation 2.9). This algorithm has been implemented on the epitope prediction server RCDEV (http://rcdev.umassmed.edu/nwpredict.php), and a similar matrix has been implemented in ProPred (http://www.imtech.res.in/raghava/propred/). For the P10 matrix, an additional column was added to the P9 matrix using values from an analysis of the specificity in the P10 pocket [72]. Values for the “Undeca” 11-mer and “Epimmune” 9-mer matrices were taken from references [24] and [48], respectively. The matrix-based IEDB [73] and Rankpep [74] predictions were performed using their respective web servers http://immuneepitope.org/home.do and http://bio.dfci.harvard.edu/RANKPEP/. Hidden Markov model HMM [29] and artificial neural network ANN [29] predictions both were performed using the Multpred web server (http://research.i2r.a-star.edu.sg/multipred/), with values for HLA-DR1 used. Potential epitopes for other HLA-DR alleles were identified using the ProPred server (http://www.imtech.res.in/raghava/propred/) [50], which implements the virtual class II matrix algorithm of Sturniolo et al. [38] for many DR alleles.

### Statistical methods.

The intrinsic predictive capabilities of the predictive algorithms were characterized using the area under the receiver operating characteristics curve (AUC ROC) [35,36]. Algorithms were compared using the AUC ROC, and product of sensitivity and specificity for each algorithm using the cutpoint at which that product is maximized. The product of sensitivity and specificity is the probability of correctly classifying a randomly selected true positive and true negative [75].

### Protein expression and purification.

For peptide binding experiments, the extracellular portion of HLA-DR1 was produced by expression of isolated subunits in Escherichia coli inclusion bodies followed by refolding in vitro as described previously [76]. Refolded HLA-DR1 was purified by immunoaffinity chromatography using the conformation-specific monoclonal antibody LB3.1, followed by gel filtration chromatography in phosphate-buffered saline (pH 6.8). The protein concentration was measured by UV absorbance at 280 nm using ɛ_280_ of 54,375 M^−1^ cm^−1^ for empty HLA-DR1.

### Peptide binding assays.

A competition assay was used to determine binding affinities of peptides to HLA-DR1 molecule. Peptide-free HLA-DR1 produced in E. coli (25 nM) was mixed together with biotinylated Ha(306–318) peptide probe (Ha_bio_, 25 nM) and varying concentrations of unlabelled competitor peptide (10^−12^ to 10^−5^ M). The mixtures were incubated for 3 d at 37 °C in 100 mM sodium phosphate buffer (pH 5.5), containing protease inhibitors and 0.5 mg/ml octylglucoside, followed by detection of bound biotinylated peptide using an immunoassay that employed anti-DR1 capture antibody LB3.1 and alkaline phosphatase-labeled streptavidin. IC_50_ values were obtained by fitting a binding curve to the plots of absorbance versus concentration of competitor peptide.

### Human donors and haplotype determination.

Nine healthy adult volunteers, three females and six males, were selected as donors for the present study. Informed consent and previous history of vaccinia immunization or infection was obtained from the donors prior to blood collection under a protocol approved by the Medical School Institutional Review Board of the University of Massachusetts. HLA class II haplotype was performed by the UMass MHC haplotyping core facility using PCR-based protocols.

### T cell lines.

TCLs were generated from blood samples obtained by venipuncture using sodium heparin as anticoagulant. Briefly, after purification by density centrifugation over Ficoll-Hypaque (Pharmacia), PBMCs were resuspended in cRPMI+10% HS (RPMI 1640 supplemented with 10% human AB+ serum, 100 U/ml penicillin, 100 μg/ml streptomycin, 1 mM sodium pyruvate, 2 mM L-glutamine, and 1 mM non-essential amino acids [GIBCO]) and 1 million cells (in 0.5 ml) were dispensed in each of six wells of a 24-well plate. Subsequently, an equal volume of a heat-inactivated lysate (60 °C for 1 h) of CV-1 cells infected with vaccinia virus in cRPMI+10% HS, originally containing 1.7 × 10^7^ pfu/ml, was added per well. After 72 h, cultures were supplemented with 1 ml of cRPMI +100 U/ml IL-2 (NCI BRB Preclinical Repository). Cells expansion proceeded for approximately 17 d in cRPMI+10% HS supplemented with 100 U/ml IL-2. TCL prepared by this method are predominately CD4^+^ (92–99% CD4^+^, see Figure S4).

### ELISPOT assay.

Recognition of peptides was evaluated by IFN-γ ELISPOT using either autologous PBMCs or EBV-B cells lines as APCs, TCLs or PBMCs as a source of T cells and peptides or a crude preparation of heat-inactivated vaccinia virus as a source of antigen. Cells and antigens, in cRPMI+10% HS, were incubated overnight (∼15 h) in plates treated as indicated by the manufacturer (BD Biosciences). Number of IFN-γ-secreting cells was determined using an ELISPOT analyzer equipped with ImmunoSpot 3.2 software (CTL analyzers). Responses were considered positive if a primary TCL showed an average >200 specific spots/million T cells (>10 spots/well over background) and a specific response at least 10-fold greater than the background response (wells without peptide) and at least 3-fold greater than the standard deviation of duplicate measurements. Weaker responses were defined as >80 specific spots/million >2-fold/background and >2 s the standard deviation of duplicate measurements.

### Depletion of CD8^+^ T cells and IFN-γ ESLIPOT.

CD8^+^ T cells were depleted from fresh PBMC samples by incubation of the samples with anti-CD8^+^ Miltenyi beads according to the manufacturer. After depletion, a sample of the CD8^+^-depleted PBMCs was removed for FACS analysis to verify the depletion. The CD8^+^-depleted cells were divided into two fractions. One of the fractions was incubated overnight with a crude preparation of heat-inactivated vaccinia virus, as described in the TCL section, and a second one in medium. The following day, non-adherent cells were recovered and assessed by IFN-γ ELISPOT using as antigen peptides as indicated before.

### Flow cytometry.

For T cell phenotype determinations, T cells were washed twice with FACS buffer (PBS + 1% BSA + 0.02% NaN_3_) and stained for 30 min with fluorescent antibodies (Pharmingen/BD Bioscience). Stained cells were washed with FACS buffer, fixed with 1% paraformaldehyde, and analyzed on a four-color BD FACSCalibur.

### Genetic restriction and EBV-B cell lines.

Genetic restriction of the T cell responses was defined by using EBV-B cell lines sharing one or more MHC molecules with the immunized DR1 donor and by inhibition of the antigen presentation with anti-MHC antibodies. For antigen presentation assays, EBV-B cells were pulsed with peptide, washed, and used as APCs in an ELISPOT assay. Cell lines (haplotypes shown in Figure S4) were obtained from the ATCC or IHWG Cell Bank (http://www.ihwg.org/cellbank/). For antibody blocking experiments, peptide-pulsed APCs were incubated with antibodies to MHC molecules. Subsequently, antibody and peptide were removed by washing and cells were used as APCs in an ELISPOT assay. Affinity-purified antibodies to MHC class I (W6/32) and DR molecules (LB3.1) were used in this study.

## Supporting Information

Figure S1 ROC Curves for Epitope Prediction Algorithms and Known HLA-DR1-Restricted T Cell Epitopes ROC curves show the tradeoff between sensitivity and specificity as the positive criterion value is varied. The diagonal line in each plot shows the ROC curve expected for a random guess. The ROC curve for a 100% accurate test would be a straight line corresponding to the left vertical axes and the top horizontal axis. Areas under the curves with standard errors are shown in Table 1.
(57 KB PDF)

Figure S2 Responses of TCLs Stimulated Twice In Vitro Recognition of vaccinia peptides by TCLs SL131 day 13, SL135, and SL136 after a second in vitro expansion with heat-inactivate lysate of CV-1 cells infected with Dryvax vaccinia virus. The bars indicate the number of specific IFN-γ producing cells by ELISPOT (mean spots in wells with peptide minus mean spots in wells without peptide) using as antigen individual peptides included in a given pool and autologous PBMCs as APCs. (A) Peptide pool 1; (B) peptide pool 2; (C) peptide pool 3; (D) peptide pool 4; (E) peptide pool 5; and (F) peptide pool 6. Background responses (wells in which peptide was not added) for donor SL131 1,023+/−72, donor SL135 23+/−12, donor SL136 37+/−24 on average per 1 × 10^6^ cells. Responses are considered positive, weak, or negative as described in Table 4.
(34 KB PDF)

Figure S3 T Cell Responses Observed in a TCL from the DR1 Donor SL131 to Peptides 301, 302, 305, 332, 334, and 335 Are Mediated by DR Molecules Inhibition of antigen presentation by antibodies to class I (W6/32) and to DR (L243). LG2 cells, sharing DRB1*0101 and DQB1*0501 with donor SL131, were pulsed with the indicated peptides and subsequently incubated on ice with antibodies to class I (gray bar) or DR (dark bars). After removal of peptide and antibody, cells were used as APCs to evaluate the response of a TCL from DR1 donor SL131 by IFN-γ ELISPOT. The values represent the percentage in the reduction of the numbers of spots, when compared to cells not treated with antibodies. The average and standard deviation number of spots in wells without antibody are: peptide 301 2,320+/−30, peptide 302 1,400+/−280, peptide 305 1,520+/−240, peptide 332 1,945+/−107, peptide 334 2,873+/−387 and peptide 335 2,640+/−244. ND, not done.
(9 KB PDF)

Figure S4 T Cell Responses Observed in a TCL from the DR1 Donor SL131 to Peptides 301, 325, and 332 Are Primarily Restricted to DR1 APCs Presentation of peptides 301, 325, and 332 by peptide-pulsed EBV-transformed B-LCLs is shown: 9273 (gray bars) sharing DRB4 and DQB1*0501; 9030 (blue bars) sharing DRB1*0407 and DRB4; 9380 (red bars) sharing DQB1*0501 and 9040 (yellow bars) do not present efficiently peptides 301, 325, and 332 to a TCL derived from donor SL131. In contrast, Hom-2 cells, sharing DR1 and DQB1*0501, are capable of antigen presentation. There is a low recognition of 325 peptide-pulsed 9273 cells, suggesting a low response mediated by either DRB4 or DQ molecules.
(41 KB PDF)

Figure S5 Phenotype of Vaccinia-Specific TCL and Analysis of PBMCs Depleted from CD8^+^ T Cells (A) Shown are representative dot plots for the phenotypic analysis of TCLs elicited to heat-inactivated vaccinia virus. T cells were washed and stained with the combination of CD3-FITC/CD4-APC antibodies, or the combination of CD3-FITC/CD8-APC antibodies. TCLs from donor SL131 day 0 (pre-boosting immunization) and day 13 (13 d post boosting immunization) are shown. TCLs from the immunized donor SL135 and the infected donor SL136 are also shown. (B) Determination of the number of CD8+ T cells in PBMCs from non-immunized donors SL139 and SL140 and from vaccinia-exposed donors SL135 and SL135 after magnetic depletion of CD8+ T cells. T cells were washed and stained with the combination of CD8-PE/CD4-APC.
(90 KB PDF)

Table S1 Low Scoring Vaccinia Peptides Accession Numbers (135 KB DOC)

Table S2 Statistical Analysis of Approaches Used to Predict Vaccinia Epitopes (39 KB DOC)

### Accession Numbers

The GenBank (http://www.ncbi.nlm.nih.gov/Genbank/) and NCBI (http://www.ncbi.nlm.nih.gov/) protein sequences used in this study are presented in Table 5. The Protein Data Bank (http://www.rcsb.org/pdb/) accession numbers are influenza virus heamagglutinin (A/New AYork/383/2004(H3N2) (1DLH), mutated TPI(23–37) from human triose phophate isomerase (1KLG), and HIV-1 strain NY-5 gag p24 protein (1SJE).

**Table 5:**
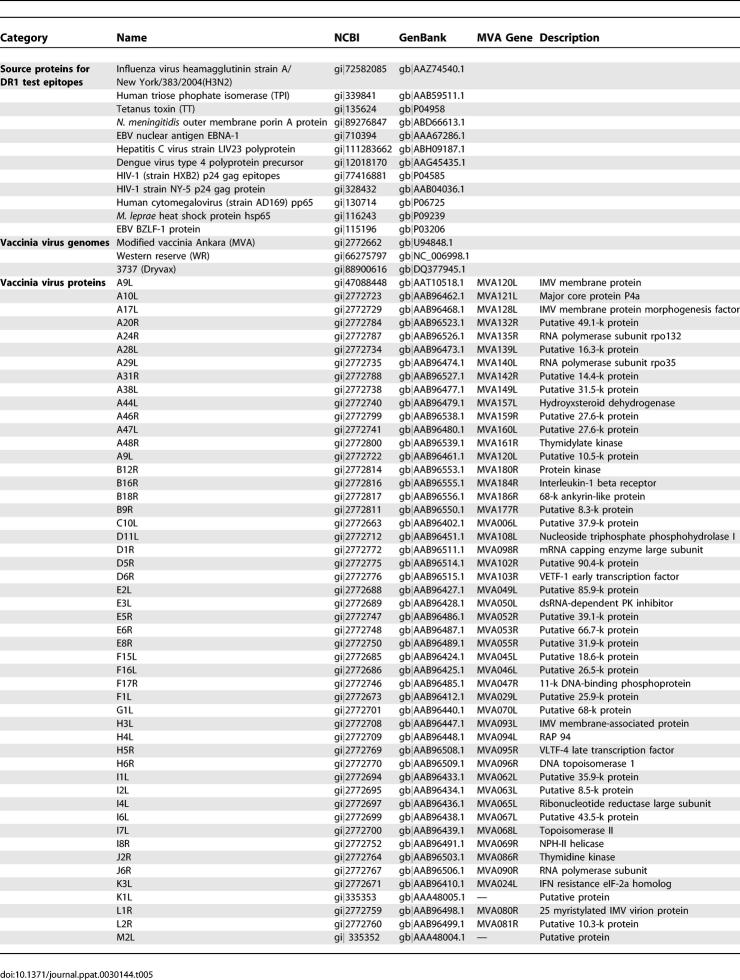
Accession Numbers

## Abbreviations

IFN-γ: interferon-γ
APC: antigen-presenting cell
DR1: DRB1*0101
EBV: Epstein–Barr virus
MHC: major histocompatibility complex
MVA: modified vaccinia Ankara
PBMC;: peripheral blood mononuclear cell isolated from whole blood
ROC: receiver-operating characteristic
TCL: T cell line
WR: Western Reserve vaccinia strain
