# Supplementary material for: Human CD4+ T Cell Epitopes from Vaccinia Virus Induced by Vaccination or Infection
Source: PLoS Pathog. 2007 Oct 12;3(10):e144. doi: 10.1371/journal.ppat.0030144 (PMC2014795; doi:10.1371/journal.ppat.0030144)
Supplement: Supplementary file 2 [file ppat.0030144.sg002.pdf]

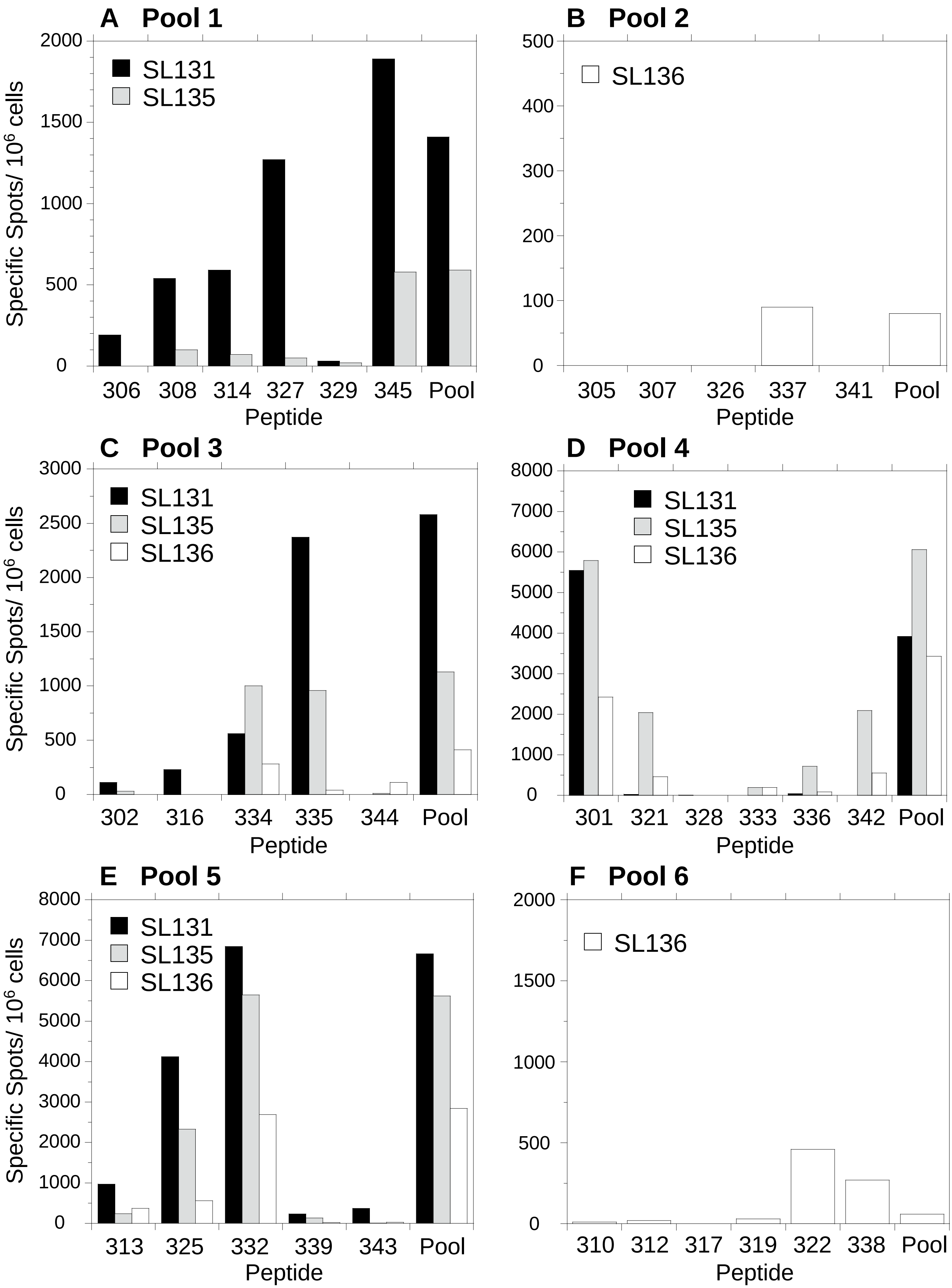

Supplemental Figure S2
