# Supplementary material for: Human CD4+ T Cell Epitopes from Vaccinia Virus Induced by Vaccination or Infection
Source: PLoS Pathog. 2007 Oct 12;3(10):e144. doi: 10.1371/journal.ppat.0030144 (PMC2014795; doi:10.1371/journal.ppat.0030144)
Supplement: Supplementary file 4 [file ppat.0030144.sg004.pdf]

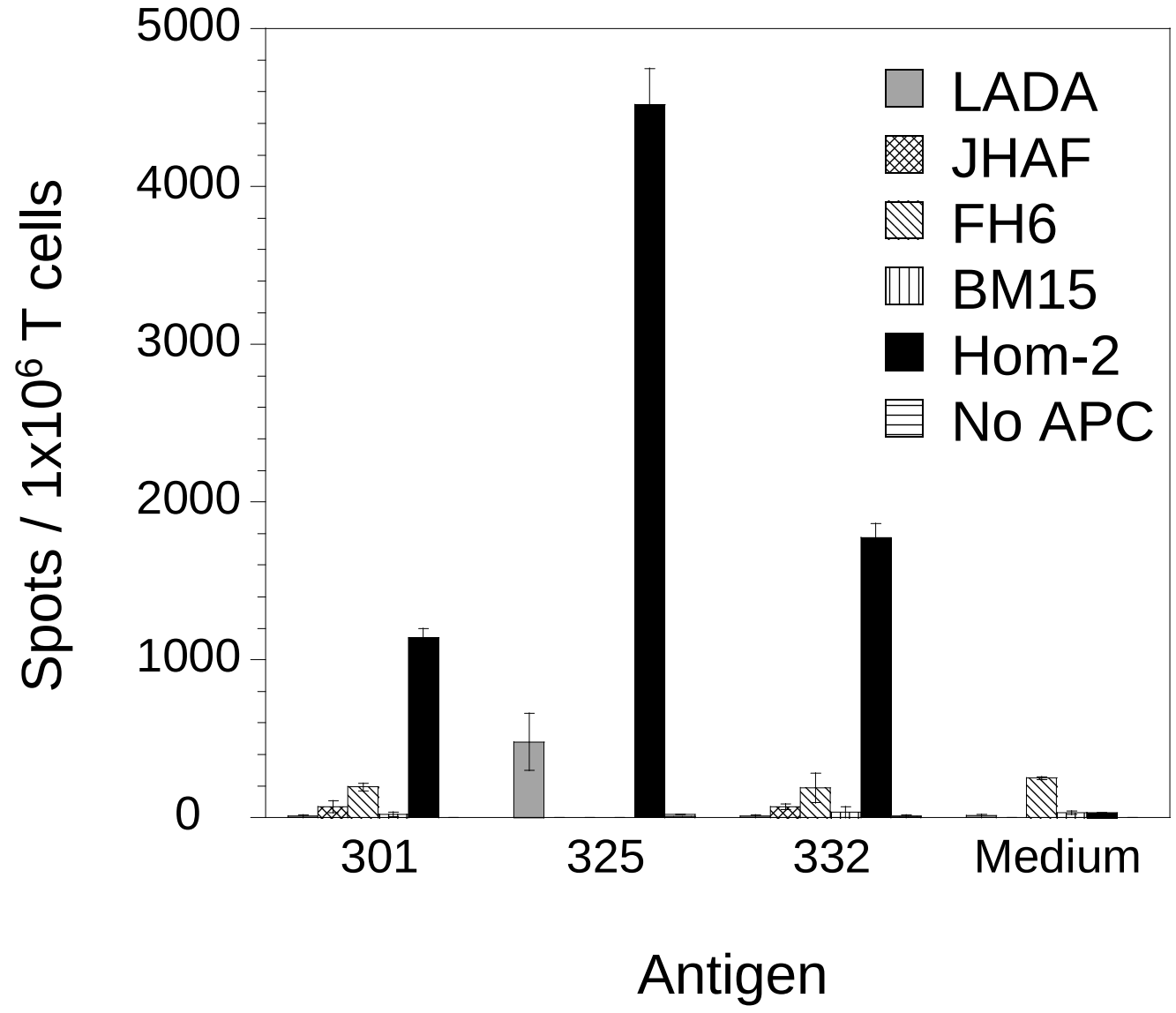

HLA CLASS II ALLELES OF APCs							T Cell Stimulation (Peptide)		
**APC**[a]	**DRB1***	**DRB3,4,5**	**DQA1***	**DQB1***	**DPA1***	**DPB1***	301	325	332
Donor SL131	**0101/0407**	**DRB4*01**	**0101/0301**	**0302/0501**	**0103/01**	**0301/0402 or 1402**	+++	+++	+++
LG2	**0101**	---	**0101**	**050101**	**0103**01	0401	+++	+++	+++
HOM-2 (9005)	**0101**	--	**0101**	**0501**	**01**	0401	+++	+++	+++
LADA (9273)	0901/1201	DRB3*02 **DRB4*01**	ND	**0501**	**0103**/0201	**0301**/1701	-	+	-
JHAF (9030)	**0407**	ND	ND	0301	ND	**0301**	-	-	-
BM15 (9040)	1102	ND	ND	0301	ND	**0301**	-	-	-
FH6 (9380)	1001/1601	DRB5*0202	ND	**0501**/0502	ND	0201/0401	-	-	-

Supplemental Figure S4
