# Supplementary material for: Human CD4+ T Cell Epitopes from Vaccinia Virus Induced by Vaccination or Infection
Source: PLoS Pathog. 2007 Oct 12;3(10):e144. doi: 10.1371/journal.ppat.0030144 (PMC2014795; doi:10.1371/journal.ppat.0030144)
Supplement: Supplementary file 6 [file ppat.0030144.st001.doc]

| Supplemental Table SI. Low scoring vaccinia peptides | | | | | | | | | | | | |  |
| --- | --- | --- | --- | --- | --- | --- | --- | --- | --- | --- | --- | --- | --- |
| Peptide  (Pool ) | Se  quence | aa | Protein of origin | Binding Prediction score for HLA DR1 | | | Expressed in Virion^a^ | | Early /Late Promoter^b^ | | | |  |
|  |  |  |  | P9 | Syfpeithi | group |  |  |  |  |  |  |  |
| B7001 (11) | DAKNN***AAKLAVDKL*** | 14 | E3L (169-182) | -3.56 | 19 | N | Y | | | E | | | |
| B7017 (11) | VIRSL***VDQLPSLPI***FDI | 17 | B18R (394-410) | -1.00 | 30 | P | nd | | | E^c^ | | | |
| **B7055** (11) | NPGKQ***VPFMRTDML*** | 14 | F17R (71-84) | -2.60 | 29 | P | Y | | | L | | | |
| COK1L01 (12) | ***LDIVKNNKL***DEIISKNKELR | 20 | K1L (255-274) | -1.35 | 26 | P | nd | | | E | | | |
| COK1L03 (12) | MD***LSRINTWKS***KQLKSFLSS | 20 | K1L (1-20) | -1.68 | 29 | P | nd | | | E | | | |
| COK1L12 (12) | DDKGNTAL***YYAVDSGNM***QTV | 20 | K1L (91-110) | -0.77 | 30 | P | nd | | | E | | | |
| COK1L14 (13) | ***KLFVKKNWRLMFYGKT***GWKT | 20 | K1L (111-130) | 0.70 | 22 | B | nd | | | E | | | |
| COK1L20 (13) | GHVD***MMILLLDYMTS***TNTNN | 20 | K1L (171-190) | -0.31 | 27 | P | nd | | | E | | | |
| COK1L27 (13) | EKHVE***YKSDSYTKDL***DIVKN | 20 | K1L (241-260) | -4.91 | 15 | N | nd | | | E | | | |
| COK1L28 (14) | YTKD***LDIVKNNKL***DEIISKN | 20 | K1L (251-270) | -1.35 | 26 | P | nd | | | E | | | |
| **COM2L03** (14) | KNTICPPRQD***YRYWYFAAEL*** | 20 | M2L (21-40) | -2.80 | 16 | N | nd | | | E | | | |
| COM2L06 (14) | TIIGE***CHMSESYIDRNANIV*** | 20 | M2L (51-70) | -0.50 | 26 | P | nd | | | E | | | |
| **COM2L09** (14) | TIMDTD***QRFVAAAEGVG***KDN | 20 | M2L (81-100) | -2.20 | 17 | N | nd | | | E | | | |
| COM2L10 (15) | AAAEGVGK***DNKLSVLLFTTQ*** | 20 | M2L (91-110) | -3.51 | 19 | N | nd | | | E | | | |
| COM2L16 (15) | ***ITINGSCVTCVNLET***DPTKI | 20 | M2L (151-170) | 1.18 | 19 | B | nd | | | E | | | |
| COM2L21 (16) | ELNQ***CLLDIKELS***YDICYRE | 20 | M2L (201-220) | -3.30 | 18 | N | nd | | | E | | | |
| MVA007 (16) | SN***YQLAVFMLM***ET | 13 | H6R (113-125) | 0.27 | 23 | B | Y | | | L^c^ | | | |
| MVA008 (16) | VN***YTFLYNFWT***NV | 13 | H6R (227- 239) | 0.70 | 20 | B | Y | | | L^c^ | | | |
| MVA013 (17) | RTR***LYDYFTRVTK*** | 13 | I1L (209-221) | -2.81 | 15 | N | Y | | | L | | | |
| MVA017 (17) | NNHG***LTPILCYIT*** | 13 | B18R (251- 263) | 0.10 | 15 | B | nd | | | E^c^ | | | |
| MVA018 (17) | IT***YLMNRFKNI***DI | 13 | B18R (310-322) | 0.84 | 20 | B | nd | | | E/L^c^ | | | |
| MVA019 (18) | IN***CLLYILRYIV***D | 13 | B18R (379-391) | 0.69 | 22 | B | nd | | | E/L^c^ | | | |
| MVA020 (18) | IRSL***VDQLPSLPI*** | 13 | B18R (395-407) | -1.00 | 30 | P | nd | | | E^C^ | | | |
| MVA025 (18) | ***YLSTERDHVMMAVRYYM*** | 17 | F1L (54-70) | 0.28 | 19 | B | nd | | | E | | | |
| MVA028 (19) | KL***MSNIKTMMI***NPFMI | 16 | A47L (81-96) | -0.71 | 28 | P | nd | | | E/L^c^ | | | |
| MVA038 (19) | TINDLKMMLF***YMDLSYHGV*** | 19 | A31R (68-86) | -0.90 | 26 | P | nd | | | E^c^ | | | |
| MVA040 (19) | NTLTE***KIFVNAFTVDK***VKQL | 20 | J6R (309-328) | 0.40 | 23 | B | Y | | | E/L | | | |
| MVA041 (20 | ***IHLLPGDWV***EVAV | 13 | J6R (358-370) | -0.30 | 33 | P | Y | | | E/L | | | |
| MVA055 (20) | KN***FTSSFYNYV***MK | 13 | B12R (33-45) | -2.10 | 19 | N | nd | | | E | | | |
| MVA057 (20) | DD***YMFFVIKNL***GR | 13 | B12R (94-106) | 0.38 | 20 | B | nd | | | E | | | |
| MVA063 (21) | SKGL***YCYFTHLGY*** | 13 | D1R (272-284) | -1.92 | 14 | N | Y | | | E | | | |
| MVA066 (21) | K***IVLYNGVNY***LNN | 13 | D1R (443-455) | 0.50 | 18 | B | Y | | | E | | | |
| MVA070 (21) | ***VEDLLSYYVVYV***F | 13 | D1R (829-841) | -1.00 | 30 | P | Y | | | E | | | |
| MVA082 (22) | ***VMCIQMKYVDI***VE | 13 | E2L (363-375) | 1.30 | 23 | B | N | | | E/L^c^ | | | |
| MVA085 (22) | KS***LDKTSLVEM***MWDL | 15 | G1L (508-522) | -4.52 | 18 | N | Y | | | L | | | |
| MVA090 (22) | VSK***LTFLDVEVF***N | 13 | A29L (155-167) | -1.40 | 26 | P | Y | | | E | | | |
| MVA092 (23) | ***FTKMAEIRA***HLKN | 13 | H5R (14-26) | -1.00 | 27 | P | Y | | | E/L | | | |
| MVA095 (23) | VLANTNF***FVFIHMVRC*** | 16 | E6R (55-70) | 2.30 | 18 | B | Y | | | E/L^c^ | | | |
| MVA096 (23) | KEYNT***YLFLHESDP***MV | 16 | E6R (417-432) | -1.00 | 27 | P | Y | | | E/L^c^ | | | |
| MVA104 (24) | ***LLNNYTLKI***SNTTFEV | 16 | A44L (299-314) | 0.34 | 23 | B | nd | | | E | | | |
| MVA105 (24) | G***LHKLKMEIF***NVE | 13 | F15L (6-18) | -2.10 | 26 | P | nd | | | E/L^c^ | | | |
| MVA142 (24) | TTK**ITPAVEYLM**D | 13 | E8R (249-261) | -2.5 | 17 | N | Y | | | E/L | | | |
| MVA143 (25) | **YLMDKLNLT**KSDVQL | 15 | E8R (258-272) | -2.1 | 28 | P | Y | | | E/L | | | |
| MVA144 (25) | G**VFMGSPEDD**LTD | 13 | I2L (010-022) | -2.2 | 18 | N | Y | | | L | | | |
| MVA157 (25) | TE**LENKKVEYV**VI | 13 | H3L (111-123) | -3.9 | 18 | N | Y | | | L | | | |
| MVA170 (26) | G**KNLQECIDF**VSE | 13 | E5R (319-331) | -2.9 | 17 | N | nd | | | E | | | |
| MVA174 (26) | RDKL**VGKTVKVKV** | 13 | K3L (055-067) | -2.5 | 27 | P | nd | | | E | | | |
| MVA185 (26) | T**IKLFNNEFD**CIR | 13 | C10L (011-023) | -2.4 | 18 | N | nd | | | E^c^ | | | |
| MVA188 (27) | YILRGL**LEAGNSLQI**FSNSV | 20 | I8R (120-139) | -1.2 | 26 | P | Y | | | E/I | | | |
| VAC2 (27) | YSND**NRYGTGLWT**HD | 15 | J2R (040-054) | -2.9 | 17 | N | nd | | | E | | | |
| VAC3 (28) | RYGTG**LWTHDKNNF**E | 15 | J2R (045-059) | -4.0 | 16 | N | nd | | | E | | | |
| VAC4 (28) | LWTHD**KNNFEALEA**T | 15 | J2R (050-064) | -3.3 | 18 | N | nd | | | E | | | |
| VAC6C (28) | TKLCDVLES | 9 | J2R (064-072) | -4.0 | 10 | N | nd | | | E | | | |
| MVA106 (29) | DN**CILANRCFV**KI | 13 | F15L (038-050) | 0.6 | 20 | B | | nd | | | E/L^c^ |  | |
| MVA186 (29) | RKIL**YRRLKNVEC** | 13 | C10L (054-066) | 3.4 | 17 | B | | nd | | | E^c^ |  | |

^A^ (Y) Found to be present in the virion or (nd) not detected by Chang et al. 2006; Resch et al. 2006; Yoder et al. 2006 [62-64].

^B^ Expected expression pattern based on promoter analysis: E, early, I, intermediate; L, late.; lower case letter indicates predicted rather than observed pattern according to Poxvirus Bioinformatics Resource Center Server ([www.poxvirus.org](http://www.poxvirus.org/))

^C^Predicted expression pattern.

Underline and bold, peptides in this set recognized by TCLs from donor SL131.
