# Supplementary material for: Human CD4+ T Cell Epitopes from Vaccinia Virus Induced by Vaccination or Infection
Source: PLoS Pathog. 2007 Oct 12;3(10):e144. doi: 10.1371/journal.ppat.0030144 (PMC2014795; doi:10.1371/journal.ppat.0030144)
Supplement: Supplementary file 7 [file ppat.0030144.st002.doc]

Supplemental Table SII Statistical analysis of approaches used to predict vaccinia

epitopes

| Test | Predicted binding P9 binding >0 | Predicted presentation Syfpeithi > 25 | Norm. Product P9*Syfpeithi^1^  ^>^ 0.55 | Norm. Sum P9+Syfpeithi^2^ > 0.75 | P9 > 0  AND  Syfpeithi > 25 |
| --- | --- | --- | --- | --- | --- |
| Sensitivity^3^ | 0.89 | 0.92 | 0.89 | 0.88 | 0.89 |
| Specificity^4^ | 0.56 | 0.48 | 0.77 | 0.75 | 0.78 |
| Positive predictive value^5^ | 0.45 | 0.42 | 0.61 | 0.58 | 0.62 |
| Sens * Spec^6^ | 0.50 | 0.45 | 0.68 | 0.66 | 0.69 |
| Youden’s J^7^ | 0.45 | 0.41 | 0.65 | 0.63 | 0.67 |
| F-measure^8^ | 0.60 | 0.58 | 0.72 | 0.70 | 0.73 |
| Area under ROC^9^ | 0.769^10^ | 0.800^10^ | 0.796^10^ | 0.800^10^ | 0.808 |

^1^ Normalized sum of the P9 and Syfpeithi scores = 0.5*(P9+16)/23 + Syfpeithi/42)

^2^ Product of the normalized P9 and Syfpeithi scores = (P9+16)/23 * Syfpeithi/42

that are correctly predicted.

^5^ Fraction of the predicted positives that actually are positive.

^6^ Product of sensitivity and specificity, provides a prevalence-independent estimate of the probability that both a positive and a negative result will be correctly predicted [75]

^7^ Sensitivity + specificity – 1 [92]

^8^ Harmonic mean of sensitivity and positive predicted value [93]

^9^ Area under the receiver-operator curve, calculated as in Table I, corresponding to the probability that a random actual positive will have a better score than a random actual negative [37]

^10^ The ROC analysis considers the entire range of scores and not just the cutoff criteria used in the analyses above.
